# Diagnostic dilemma in Cushing’s syndrome: discrepancy between patient-reported and physician-assessed manifestations

**DOI:** 10.1007/s12020-024-03935-9

**Published:** 2024-06-25

**Authors:** Yuma Motomura, Shin Urai, Hironori Bando, Masaaki Yamamoto, Masaki Suzuki, Naoki Yamamoto, Genzo Iguchi, Wataru Ogawa, Hidenori Fukuoka

**Affiliations:** 1 Division of Diabetes and Endocrinology, Department of Internal Medicine, Kobe University Graduate School of Medicine, Kobe, Hyogo 650-0017 Japan; 2 Division of Diabetes and Endocrinology, Department of Internal Medicine, Kobe University Hospital, Kobe, Hyogo 650-0017 Japan; 3 Medical Center for Student Health, Kobe University, Kobe, Hyogo 657-8501 Japan; 4 Department of Biosignal Pathophysiology, Kobe University Graduate School of Medicine, Kobe, Hyogo 657-8501 Japan

**Keywords:** Cushing’s syndrome, Cortisol, Patients’ recognition, Early diagnosis

## Abstract

**Purpose:**

Early diagnosis and immediate treatment of Cushing’s syndrome (CS) are critical for a better prognosis but remain a challenge. However, few comprehensive reports have focused on this issue or investigated whether patient-reported manifestations are consistent with physician-assessed symptoms of CS. This study aimed to clarify the differences in patient-reported and physician-assessed manifestations of signs and symptoms of CS that prevent early diagnosis.

**Methods:**

This single-center retrospective study included 52 patients with CS (16 with Cushing’s disease and 36 with adrenal CS). Upon clinical diagnosis, medical records were used to independently review the patient-reported and physician-assessed manifestations of typical (such as purple striae and proximal myopathy) and nonspecific features (such as hirsutism and hypertension). The correlations and differences between the patient-reported and physician-assessed manifestations were then analyzed.

**Results:**

We observed a positive correlation between the total number of manifestations of nonspecific features reported by patients and those assessed by physicians, but not for typical features. Moreover, manifestations reported by the patients were less frequent than those assessed by physicians for typical features, leading to discrepancies between the two groups. In contrast, there were no differences in most nonspecific features between the patient-reported and physician-assessed manifestations. Notably, the concordance between patient-reported and physician-assessed manifestations of typical features was not associated with urinary free cortisol levels.

**Conclusion:**

Regardless of disease severity, patients often do not complain of the typical features of CS that are crucial for formulating a diagnosis.

## Introduction

Endogenous Cushing’s syndrome (CS) is caused by chronic and excessive glucocorticoid exposure. This occurs primarily due to adrenocorticotropic hormone (ACTH)-producing pituitary tumors (Cushing’s disease; CD) or cortisol-producing adrenal tumors (adrenal Cushing’s syndrome; ACS) [1]—and has a high mortality rate owing to cardiovascular disease, severe infection, and suicide, even when diagnosed and treated appropriately [1, 2]. Moreover, the prognosis is poor if the disease is not adequately treated or remains undiagnosed [2]. Therefore, early diagnosis and immediate intervention are important, as remission of CS due to surgical and pharmacological treatment can reduce the risk of mortality [3, 4].

CS is a rare disease with a prevalence of 57 per million individuals and an annual incidence of 3.2 per million, and its epidemiology is consistent across various regions worldwide [5, 6]. Most symptoms and signs of CS are common in general metabolic disorders, including obesity, hypertension, osteoporosis, and diabetes mellitus [7]. However, CS should be suspected if these symptoms appear as unusual features for their age [1, 8]. Consequently, the identification of CS is challenging and labor-intensive [1, 9, 10]. In fact, recent research revealed that a definitive diagnosis of CD (the most common form of CS), took an average of 3.8 ± 4.8 years from the onset of symptoms, and patients typically consulted 4.6 ± 3.8 medical professionals before this disease was identified [11]. Typical features of CS include symptoms of moon face, central obesity, or buffalo hump [12], which are similar to other symptoms such as primary obesity and therefore can lead to misdiagnosis. Furthermore, although purple striae or thin skin with an increased propensity for bruising are other typical features of CS [12], these attributes are not commonly acknowledged by the general population [1, 9].

Attempts have been made to diagnose CS early, including the development of scoring systems to estimate the pre-test probability of CS and facial image analysis software to diagnose the specific facial features of CS [13–15]; however, these have not yet been used widespread or fully and the early diagnosis of CS remains dependent on the experience-based medical skills of the clinical staffs [16].

Additionally, although it is difficult for patients to recognize complex and nonspecific symptoms [17, 18], the significance of patients recognizing their illness has recently been reported for various diseases such as heart failure and malignant carcinoma [19–21]. It is widely acknowledged that patients’ self-recognition can result in early detection of the disease, reduce its severity and recurrence, and enhance their quality of life [19]. In patients with endocrine diseases, there is increasing focus on issues surrounding self-recognition [22–24]. For example, a previous study focusing on acromegaly reported a discrepancy between patient-reported and physician-reported manifestations and indicated that resolving this discrepancy could shorten the time to diagnosis [25].

Identifying CS may be challenging for primary care physicians who are yet to specialize. Therefore, endocrinologists with extensive experience in CS have often noticed that patients and these physicians struggle to identify the symptoms of CS; however, few comprehensive reports have focused on this issue or investigated whether patient-reported manifestations are consistent with physician-assessed symptoms of CS.

Therefore, this study aimed to investigate the unreported manifestations of CS among individuals referred to non-specialist healthcare providers, including primary care physicians, and to recognize potential challenges with the current diagnosis of CS with the goal of facilitating early detection.

## Materials and methods

### Patients, study design, and data collection

This single-center retrospective study was conducted to identify the discrepancies between patient-reported and physician-assessed symptoms and investigate the factors causing these differences.

From September 2004 to December 2022, 199 patients were referred to our department at a tertiary medical institution upon suspicion, evaluation, or follow-up for hypercortisolism. Of these patients, 92 were newly diagnosed with CS (36 with CD, 51 with ACS, and 5 with ectopic ACTH syndrome) based on the diagnostic guidelines [3, 8, 12], with a diagnosis confirmed by pathological evaluation after surgical resection [26]. However, 35 patients were excluded due to a lack of detailed clinical data on the manifestations at diagnosis. Similarly, we excluded individuals diagnosed with ectopic ACTH syndrome because of the lack of comprehensive information on symptoms reported by the patients and primary care physicians due to the rapid progression and severity of this disease. Therefore, 52 patients (16 with CD and 36 with ACS) were enrolled in this study.

Upon clinical diagnosis, the manifestations included in the comprehensive standardized interview at the time of diagnosis and those assessed by the physician through collaborative assessment with multiple board-certified endocrinologists as routine practice were independently reviewed from the medical records. We categorized these manifestations reviewed from the medical records into the following two categories based on the diagnostic guidelines including those of the Japan Endocrine Society: typical features, including moon face, central obesity or buffalo hump, purple striae of ≥1 cm, thin skin and easy bruising, and proximal myopathy; and nonspecific features (shown as atypical in Japan Endocrine Society’s guideline), including hypertension, menstrual abnormalities, acne, hirsutism, peripheral edema, glucose metabolism impairment, osteoporosis, pigmentation (which is not expected in patients with ACS), and mental abnormalities [1, 8, 12]. Central obesity or buffalo hump can also be observed in pseudo CS. However, in this study, features were classified as the same typical feature according to clinical guidelines [12, 27]. We also reviewed the biochemical findings, comorbidities, duration from the initial recognition of CS-related symptoms to diagnosis, and number of medical institutions visited before diagnosis.

The present retrospective study was performed in accordance with the Declaration of Helsinki and approved by the Ethics Committee of Kobe University Hospital (Approval No. 1351). The patients had the option of an opt-out process, and all procedures were part of routine medical care.

### Definition of patient-reported and physician-assessed manifestations

In the context of routine clinical care, physicians asked the patients about the presence or absence of manifestations and comorbidities (e.g., hypertension, menstrual abnormalities, glucose metabolism impairment, osteoporosis, and mental abnormalities), which were documented in the medical records. These reports in the medical records were defined as patient-reported manifestations in this study. In contrast, the manifestations and comorbidities of CS were assessed within several weeks after the patient was referred to our department for suspected CS. Additional diagnostic information on comorbidities is provided in the subsequent section. Physician-assessed manifestations were subsequently defined based on these findings.

### Comorbidities of Cushing’s syndrome

All comorbidities were diagnosed according to the appropriate guidelines [28–30]. For example, hypertension was diagnosed if patients were taking oral antihypertensive medication or had more than grade 1 hypertension (≥140/90 mmHg) in a treatment-naïve state [28]. Moreover, glucose metabolism impairment—including diabetes mellitus, impaired glucose tolerance, and impaired fasting glucose—was diagnosed based on the results of blood glucose levels during fasting and after a 75-g oral glucose tolerance test, as well as hemoglobin A1c (HbA1c) levels [29]. Patients taking medications for diabetes mellitus at the time of CS diagnosis were also categorized as having diabetes.

Other comorbidities included mental abnormalities, menstrual abnormalities, and the presence of osteoporosis. Mental abnormalities were defined as the use of anxiolytic medications, sleeping pills, or antidepressants prescribed by experienced psychologists, and menstrual abnormalities were defined as women with irregular menstrual cycles. Furthermore, the presence of osteoporosis was defined as bone mineral density (BMD) of <–2.5 standard deviations (SD) of the T-score of the lumbar vertebrae (L2–L4), femoral neck, or distal radius measured using dual-energy x-ray absorptiometry (DXA; Horizon A DXA System), and/or an experience of a fragility fracture [30]. As per the specifications of the measurement system employed, L1 was not included in the assessment. The Z-score was also employed as a diagnostic reference among young adults. Patients also diagnosed with osteoporosis who were receiving medications for this disease.

### Hormone assay

In this study, blood samples were collected after an overnight fast. Subsequently, serum cortisol levels were measured using a chemiluminescent enzyme immunoassay [CLEIA] (TOSOH, Tokyo, Japan, RRID:AB_3099658) or enzyme immunoassay [EIA] (TOSOH, Tokyo, Japan, RRID:AB_3076600). Similarly, plasma ACTH levels were measured using a CLEIA (TOSOH, Tokyo, Japan, RRID:AB_3099657, or Siemens, Tokyo, Japan, RRID:AB_2909441) and EIA (TOSOH, Tokyo, Japan, RRID:AB_2783633). In both methods, the measurements showed good correlation and no conversion was required [31, 32].

Urinary free cortisol (UFC) levels were also measured using radioimmunoassays (RIA; TFB, Tokyo, Japan, RRID:AB_2894408) or chemiluminescent immunoassays (CLIA; Siemens, Tokyo, Japan, RRID:AB_2893154). Using the following formula, the UFC levels measured by RIA were then corrected to the value measured by CLIA: Y = 0.832X − 4.23 (Y = UFC levels using CLIA, X = UFC levels using RIA) [33].

### Statistical analysis

All statistical analyses were performed using SPSS ver. 28.0 software (IBM Corp., Armonk, NY, USA). All continuous variables were analyzed using the Shapiro–Wilk normality test to confirm a normal distribution, whereas Fisher’s exact test was used to analyze categorical data. Between the two groups, differences in normally or non-normally distributed data were compared using the unpaired Student’s *t*-test or the Mann–Whitney *U* test, respectively.

Cohen’s kappa coefficient was used to describe the concordance between the patient-reported and physician-assessed manifestations. As previously reported [19, 20, 34], the concordance based on the value of Cohen’s kappa coefficient was rated as follows: 0.00–0.20 for “Slight,” 0.21–0.40 for “Fair,” 0.41–0.60 for “Moderate,” 0.61–0.80 for “Substantial,” and 0.81–1.00 for “Almost Perfect.” For correlation analysis between two variables of non-normally distributed data, we used Spearman’s rank correlation coefficient. Multivariate logistic regression analyses were then performed to investigate variables associated with the discrepancies between patient-reported and physician-assessed manifestations.

The results are presented as mean ± SD for normally distributed data and median [interquartile range] for non-normally distributed data, and differences were considered statistically significant when the *P* value was <0.05.

## Results

### Clinical characteristics of the patients

We included 52 patients diagnosed with CS in this study. Their clinical characteristics are presented in Table 1. Notably, this group consisted of 5 males and 47 females, with a mean age of 49.4 ± 15.8 years, median body mass index (BMI) of 23.0 [21.3–28.0] kg/m^2^, and median UFC level of 272.1 [126.0–435.0] µg/day. Of the CS patients, 16 had CD and 36 had ACS, which is consistent with epidemiological data on CS observed in Asians (including Japanese individuals); however, this differed from epidemiological data from Western countries [35, 36]. Regarding comorbidities, 43 patients were diagnosed with hypertension—of which 34 were prescribed antihypertensive medications—with a mean systolic blood pressure (BP) of 136.4 ± 21.5 mmHg and diastolic BP of 83.5 ± 15.0 mmHg. In addition, 44 patients were diagnosed with glucose metabolism impairment—of which, 20 were prescribed oral hypoglycemic agents and/or insulin—with a median fasting serum glucose level of 99.5 [87.3–116.5] mg/dL and median HbA1c level of 6.3% [5.7–7.4]. Moreover, 29 patients were diagnosed with osteoporosis, of which 4 were prescribed antiosteoporosis medication, with BMD T-score SDs of -1.54 ± 1.39, -1.76 ± 1.12, and -0.50 [-1.53–0.50] for the lumber spine, femoral neck, and distal radius, respectively. Notably, the UFC levels were higher in patients with CD than in those with ACS (412.6 [243.2–1,100.3] *vs*. 215.3 [114.0–387.8] µg/day); however, there were no significant differences attributed to sex, age, BMI, or the proportion of patients with respect to comorbidities, including hypertension and glucose metabolism impairment, between patients with CD and ACS.

**Table 1:** Clinical characteristics of the patients

	Total	CD	ACS	CD vs. ACS *P* value
Number of men/women	5/47	1/15	4/32	1.00
Age (years)	49.4 ± 15.8	54.3 ± 19.2	47.2 ± 13.8	0.14
BMI (kg/m^2^)	23.0 [21.3–28.0]	24.7 [22.2–30.0]	22.8 [20.8–26.4]	0.17
Midnight F (µg/dL)	20.1 [16.0–23.5]	20.2 [13.9–24.7]	20.1 [16.9–23.0]	0.97
F after LDDST (μg/dL)	21.2 ± 6.9	24.2 ± 10.1	19.7 ± 4.2	0.11
UFC (μg/day)	272.1 [126.0–435.0]	412.6 [243.2–1,100.3]	215.3 [114.0–387.8]	0.02
Basal ACTH (pg/mL)	2.0 [0.0–53.9]	83.2 [57.4–169.9]	0.0 [0.0–2.1]	<0.01
Systolic BP (mmHg)	136.4 ± 21.5	140.5 ± 20.7	134.6 ± 21.8	0.36
Diastolic BP (mmHg)	83.5 ± 15.0	83.1 ± 14.3	83.6 ± 15.5	0.90
Use of antihypertensive drugs, n (%)	34 (65)	13 (81)	21 (58)	0.13
FSG (mg/dL)	99.5 [87.3–116.5]	110.0 [102.0–142.8]	92.5 [83.3–114.3]	0.01
HbA1c (%)	6.3 [5.7–7.4]	6.8 [5.9–8.6]	6.0 [5.7–7.1]	0.08
Use of OHA and/or insulin, n (%)	20 (38)	9 (56)	11 (31)	0.12
LS BMD T-score (SD)	−1.54 ± 1.39	−1.00 ± 1.38	−1.79 ± 1.35	0.07
LS BMD Z-score (SD)	−0.78 ± 1.37	0.13 ± 1.11	−1.20 ± 1.28	<0.01
FN BMD T-score (SD)	−1.76 ± 1.12	−1.73 ± 1.54	−1.78 ± 0.88	0.92
FN BMD Z-score (SD)	−0.79 ± 1.01	−0.39 ± 1.10	−0.99 ± 0.92	0.10
Radius BMD T-score (SD)	−0.50 [−1.53–0.50]	−0.30 [−2.50–0.40]	−0.60 [−1.30–0.60]	0.79
Radius BMD Z-score (SD)	0.60 [−0.60–1.50]	1.50[−0.60–1.80]	0.50[−0.50–1.00]	0.33
Use of antiosteoporosis drugs, n (%)	4 (8)	1 (6)	3 (8)	1.00
Time to diagnosis (months)	44.0 [13.3–125.3]	43.0 [15.0–128.3]	47.5 [12.5–125.3]	0.87
Number of medical institutions before diagnosis	3.0 [2.0–5.0]	3.0 [2.0–5.0]	3.0 [3.0–5.8]	0.23

The results are presented as mean ± SD for normally distributed data and median [interquartile range] for non-normally distributed data

*CD* Cushing’s disease, *ACS* adrenal Cushing’s syndrome, *BMI* body mass index, *F* cortisol, *LDDST* low-dose dexamethasone suppression test, *UFC* urinary free cortisol, *ACTH* adrenocorticotropic hormone, *BP* blood pressure, *FSG* fasting serum glucose, *HbA1c* hemoglobin A1c, *OHA* oral hypoglycemic agents, *BMD* bone mineral density, *LS* lumber spine, *FN* femoral neck

The median duration from the patients’ initial recognition of CS-related manifestations to diagnosis was 44.0 [13.3–125.3] months, and it took more than 3 years to diagnose CS in 30 patients (58%). Furthermore, the median number of medical facilities visited by patients before diagnosis was 3.0 [2.0–5.0]; however, there were no significant differences in the duration or number of medical institutions between patients with CD and those with ACS.

### Frequency and concordance between patient-reported and physician-assessed CS-related manifestations

**Fig. 1:**
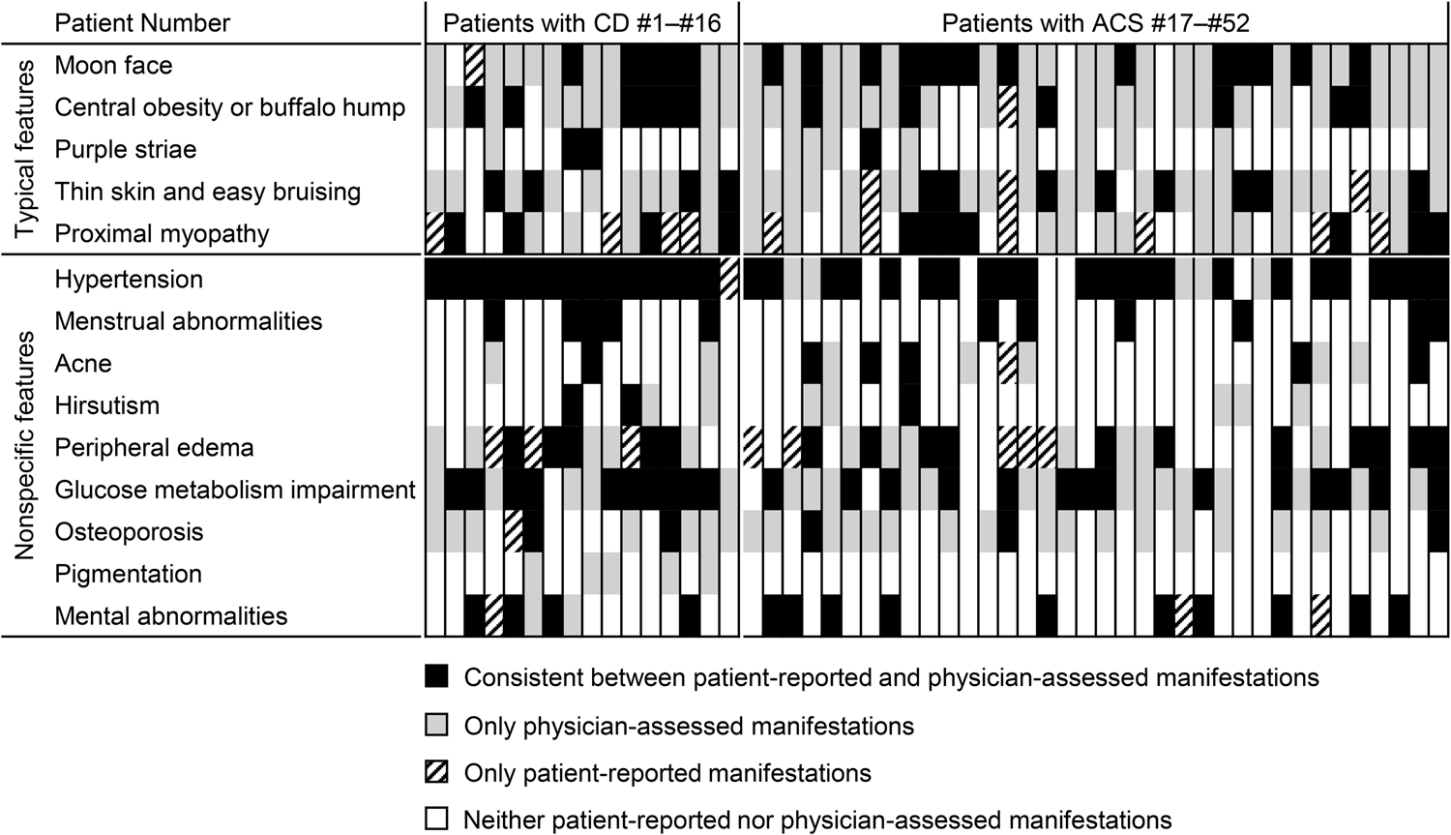
Consistency between patient-reported and physician-assessed manifestations for each individual case. The consistencies or discrepancies between patient-reported and physician-assessed manifestations are shown. Vertical lines represent manifestations in individual patients. CD Cushing’s disease, ACS adrenal Cushing’s syndrome

Each manifestation reported by a patient or assessed by a physician is shown vertically for individual cases in Fig. 1. Compared with nonspecific features, typical features appeared to not be reported by the patients but were only assessed by the physicians. In addition, compared to nonspecific features, there were fewer cases in which the manifestations reported by the patients were consistent with those assessed by physicians for typical features.

**Fig. 2:**
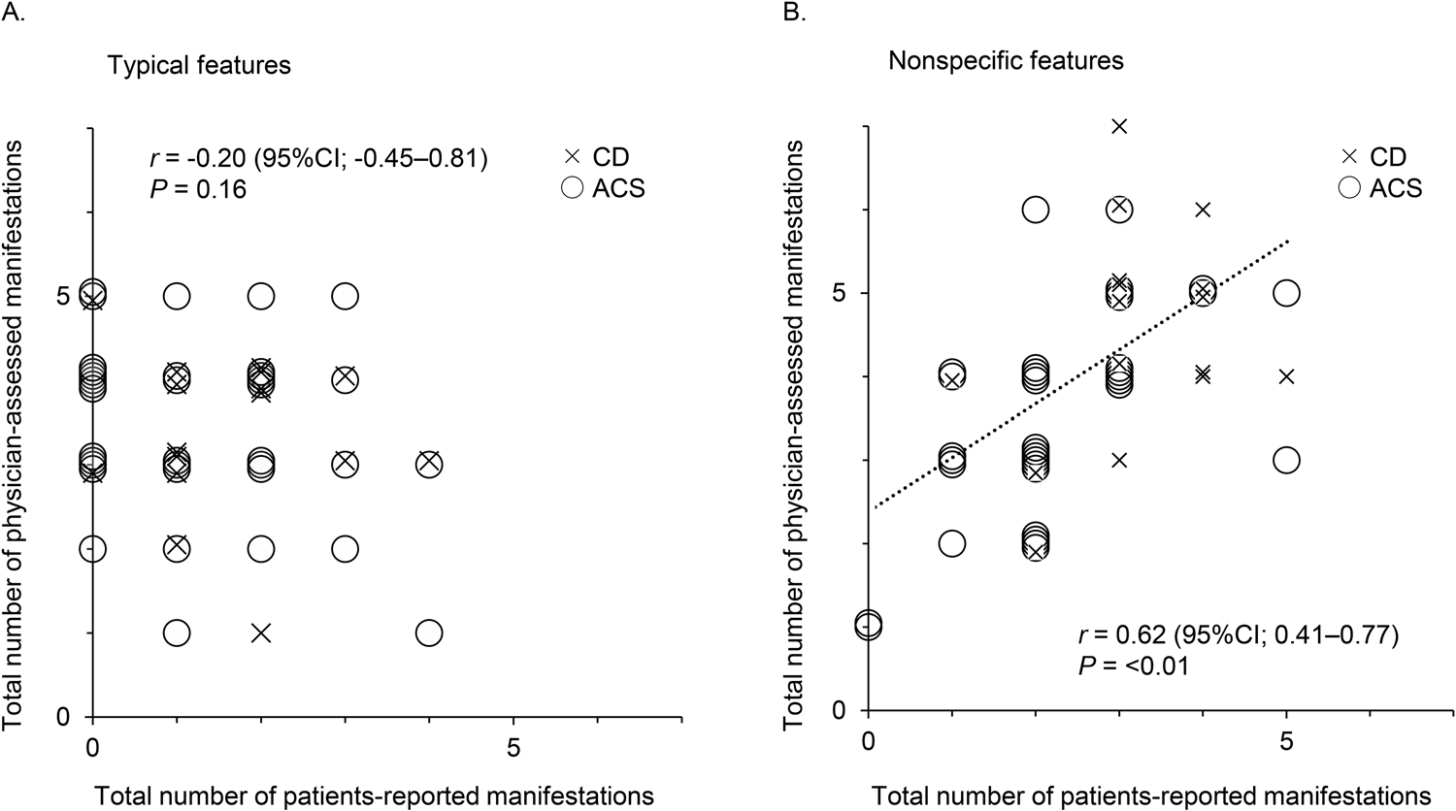
Correlation between the total number of patient-reported and physician-assessed manifestations. Correlations between the total number of patient-reported and physician-assessed manifestations are shown for typical (**A**) and nonspecific features (**B**). CD is plotted by ×, and ACS is plotted by ○. The Spearman’s rank correlation coefficients and *P* value are presented. CI confidence interval, CD Cushing’s disease, ACS adrenal Cushing’s syndrome

Consistent with the impact of these visually distinctive presentations shown in Fig. 1, no correlation was observed in the number of typical features between patient-reported and physician-assessed manifestations (*r* = –0.20, *P* = 0.16) (Fig. 2A), whereas a positive correlation was found for nonspecific features (*r* = 0.62, *P* < 0.01) (Fig. 2B). Moreover, the total number of patient-reported manifestations of typical features was lower than that of physician-assessed manifestations (1.0 [0.0–2.0] *vs*. 3.5 [3.0–4.0], *P* < 0.01), and four of the five typical features were reported less frequently by patients than by physicians, except for proximal myopathy (Table 2A). According to Cohen’s kappa coefficient, the concordance between patient-reported and physician-assessed manifestations was marked as “Fair” to “Slight,” indicating a discrepancy for all typical features. Similarly, the total number of patient-reported manifestations of nonspecific features was also lower than that in physicians (2.5 [2.0–3.0] *vs*. 4.0 [3.0–5.0], *P* < 0.01). However, except for glucose metabolism impairment or osteoporosis, there were no differences in the frequencies of nonspecific features between patient-reported and physician-assessed manifestations, and the concordance of the nonspecific features between the patient-reported and physician-assessed manifestations was “Almost perfect” for menstrual abnormality and “Substantial” for mental abnormality and hypertension, whereas that for glucose metabolism impairment and osteoporosis was “Fair.” This suggests that the discrepancy between patient-reported and physician-assessed manifestations was more significant for typical than for nonspecific features. However, no differences in these discrepancies were observed between patients with CD and those with ACS (Table 2B, C).

**Table 2:** Frequencies of patient-reported and physician-assessed manifestations and their concordance. **A**. All patients (*n* = 52). **B**. Patients with CD (*n* = 16). **C**. Patients with ACS (*n* = 36)

	Patient-reported	Physician-assessed	*P* value of Fisher’s exact test	Concordance with Cohen’s kappa coefficient
**A**				
**Typical features**				
Moon face, n (%)	20 (39)	48 (92)	<0.01	Slight
Central obesity or buffalo hump, n (%)	13 (25)	44 (85)	<0.01	Slight
Purple striae, n (%)	3 (6)	15 (29)	<0.01	Fair
Thin skin and easy bruising, n (%)	15 (29)	43 (83)	<0.01	Slight
Proximal myopathy, n (%)	21 (40)	27 (52)	0.33	Slight
**Nonspecific features**				
Hypertension, n (%)	39 (75)	43 (83)	0.47	Substantial
Menstrual abnormalities, n (%)	11 (21)	11 (21)	1.00	Almost perfect
Acne, n (%)	7 (14)	13 (25)	0.21	Moderate
Hirsutism, n (%)	3 (6)	10 (19)	0.07	Moderate
Peripheral edema, n (%)	24 (46)	28 (54)	0.56	Fair
Glucose metabolism impairment, n (%)	24 (46)	44 (85)	<0.01	Fair
Osteoporosis, n (%)	7 (14)	29 (56)	<0.01	Slight
Pigmentation, n (%)	0 (0)	5 (10)	0.06	-
Mental abnormalities, n (%)	17 (33)	17 (33)	1.00	Substantial
**B**				
**Typical features**				
Moon face, n (%)	6 (38)	14 (88)	0.01	Slight
Central obesity or buffalo hump, n (%)	6 (38)	15 (94)	<0.01	Slight
Purple striae, n (%)	2 (13)	4 (25)	0.56	Moderate
Thin skin and easy bruising, n (%)	4 (25)	13 (81)	0.06	Slight
Proximal myopathy, n (%)	8 (50)	8 (50)	1.00	Slight
**Nonspecific features**				
Hypertension, n (%)	16 (100)	15 (94)	0.78	Slight
Menstrual abnormalities, n (%)	5 (31)	5 (31)	1.00	Almost perfect
Acne, n (%)	1 (6)	3 (19)	0.56	Moderate
Hirsutism, n (%)	2 (13)	4 (25)	0.56	Moderate
Peripheral edema, n (%)	8 (50)	10 (63)	0.56	Slight
Glucose metabolism impairment, n (%)	10 (63)	15 (94)	0.14	Slight
Osteoporosis, n (%)	4 (25)	9 (56)	0.15	Slight
Pigmentation, n (%)	0 (0)	5 (31)	0.14	-
Mental abnormalities, n (%)	5 (31)	6 (38)	0.78	Moderate
**C**				
**Typical features**				
Moon face, n (%)	14 (39)	34 (94)	<0.01	Slight
Central obesity or buffalo hump, n (%)	7 (19)	29 (81)	<0.01	Slight
Purple striae, n (%)	1 (3)	11 (31)	<0.01	Slight
Thin skin and easy bruising, n (%)	11 (31)	30 (83)	<0.01	Slight
Proximal myopathy, n (%)	13 (36)	19 (53)	0.24	Slight
**Nonspecific features**				
Hypertension, n (%)	23 (64)	28 (78)	0.30	Substantial
Menstrual abnormalities, n (%)	6 (17)	6 (17)	1.00	Almost perfect
Acne, n (%)	6 (17)	10 (28)	0.40	Moderate
Hirsutism, n (%)	1 (3)	6 (17)	0.11	Fair
Peripheral edema, n (%)	16 (44)	18 (50)	0.81	Fair
Glucose metabolism impairment, n (%)	14 (39)	29 (81)	<0.01	Fair
Osteoporosis, n (%)	3 (8)	20 (56)	<0.01	Slight
Pigmentation, n (%)	0 (0)	0 (0)	-	-
Mental abnormalities, n (%)	12 (33)	11 (31)	1.00	Almost perfect

The frequencies of patient-reported and physician-assessed manifestations were compared using Fisher’s exact test. The concordance between patient-reported and physician-assessed manifestations was evaluated with Cohen’s kappa coefficient, and its coefficients were defined as follows: 0.00–0.20 for “Slight,” 0.21–0.40 for “Fair,” 0.41–0.60 for “Moderate,” 0.61–0.80 for “Substantial,” and 0.81–1.00 for “Almost perfect”

*CD* Cushing’s disease, *ACS* adrenal Cushing’s syndrome

We performed logistic regression analyses using UFC to investigate whether excess cortisol levels influenced the discrepancy between patient-reported and physician-assessed manifestations. Notably, we observed no association between UFC levels and discrepancies between patient-reported and physician-assessed manifestations in the univariate or multivariate logistic regression analyses adjusted for sex and age (Table 3A). In addition, no association was observed after adjusting for other variables such as BMI and disease duration. Similarly, we found that the serum cortisol levels after the low-dose dexamethasone suppression test (LDDST) were not associated with discrepancies between patient-reported and physician-assessed manifestations (Table 3B). Thus, these disparities were shown to be insignificant when directly related to the severity of CS.

**Table 3:** Logistic regression analyses of the discrepancies between the patient-reported and physician-assessed manifestations. **A**. Variables associated with UFC levels. **B**. Variables associated with serum cortisol levels after the LDDST

	Univariate	Multivariate 1 (sex- and age-adjusted)	Multivariate 2 (BMI-adjusted)	Multivariate 3 (disease duration-adjusted)
**A**				
Moon face	1.000 (0.999–1.001)	1.000 (0.999–1.001)	1.000 (0.998–1.002)	1.000 (0.999–1.001)
Proximal myopathy	1.000 (0.999–1.001)	1.000 (0.999–1.001)	1.000 (0.998–1.001)	1.000 (0.998–1.001)
Thin skin and easy bruising	1.000 (0.998–1.001)	1.000 (0.999–1.001)	1.000 (0.999–1.001)	1.000 (0.998–1.001)
Central obesity or buffalo hump	1.001 (1.000–1.003)	1.001 (1.000–1.003)	1.001 (1.000–1.003)	1.001 (1.000–1.003)
Purple striae	1.000 (0.999–1.002)	1.000 (0.998–1.002)	1.001 (0.999–1.003)	1.000 (0.999–1.002)
**B**				
Moon face	0.998 (0.919–1.084)	0.999 (0.919–1.086)	1.000 (0.920–1.088)	0.997 (0.918–1.082)
Proximal myopathy	1.007 (0.925–1.097)	1.007 (0.924–1.097)	1.007 (0.925–1.097)	1.006 (0.924–1.096)
Thin skin and easy bruising	1.022 (0.939–1.112)	1.018 (0.934–1.109)	1.023 (0.940–1.113)	1.019 (0.937–1.109)
Central obesity or buffalo hump	0.979 (0.890–1.078)	0.978 (0.865–1.105)	0.981 (0.875–1.099)	0.978 (0.887–1.078)
Purple striae	0.998 (0.919–1.084)	0.999 (0.919–1.086)	1.000 (0.920–1.088)	0.997 (0.918–1.082)

The results are presented as odds ratios (95% confidence intervals)

*UFC* urinary free cortisol, *BMI* Body Mass Index, *LDDST* low-dose dexamethasone suppression test

## Discussion

In the present study, we highlight the challenges associated with the diagnosis of CS—a condition resulting from excessive glucocorticoid exposure—and elucidate the divergence between patient-reported and physician-assessed manifestations. Thus, this study may aid in the early detection of CS by identifying symptoms that patients are unable to recognize based on the disparities between patient-reported and physician-assessed manifestations of CS.

In this study, the number of patient-reported manifestations of both typical and nonspecific features was lower than that of physician-assessed manifestations, suggesting that CS symptoms may have been overlooked by relying solely on patient reports. Additionally, analysis of the concordance between patient-reported and physician-assessed manifestations revealed a tendency for these manifestations to be inconsistent for both typical and nonspecific features, with a tendency to be more significant for typical features. Furthermore, the UFC and serum cortisol levels after the LDDST, which represent the severity of CS, were not associated with the concordance of manifestations between patients and physicians, suggesting that even in cases of severe CS, patients may not recognize their symptoms. These findings imply that typical features, which are essential for diagnosing CS, may be difficult for patients to recognize and poorly identified or conveyed to patients by non-specialist physicians, who are typically the first to interact with individuals with CS. The importance of educating healthcare providers such as primary care physicians, family physicians and gynecologists for early diagnosis of CS should be highlighted.

According to a previous report on the diagnostic history of 176 patients with CD, 83% of the patients visited their family physician for manifestations such as weight gain and hypertension, while 46% visited a gynecologist for menstrual abnormalities before the diagnosis of CD [11]. Thus, the typical features of CS were not recognized. The examination may reveal nonspecific features. However, individuals who are non-specialists may not recognize these features as indications of CS. Therefore, patients are often unaware of the potential complications associated with CS. This is consistent with the results of our study, in which patient-reported and physician-assessed manifestations were more consistent for hypertension and menstrual abnormalities than for other manifestations such as typical features, glucose metabolism impairment, and osteoporosis. This makes diagnosis challenging as non-specialist physicians and, more prominently, patients may not recognize the full range of symptoms associated with CS, especially the typical features with high diagnostic value. In addition, older patients diagnosed with CS present with a lower BMI and waist circumference than younger patients [37], and they typically do not exhibit symptoms commonly associated with CS such as skin alterations, depression, hair loss, hirsutism, and reduced libido. These findings may further complicate the diagnosis of CS in elderly patients.

By evaluating only the patient-reported manifestations, it appears that manifestations such as peripheral edema and proximal myopathy were more common. Possibly, these symptoms were not considered features of CS by physicians, in comparison to the degree of symptoms experienced by the patients. However, this may not necessarily imply diminishing the significance of the patient’s signs and symptoms, as these manifestations can be considered as the unidentified complaints and may result in a postponement of the diagnosis of CS. Patients may be experiencing symptoms that physicians do not perceive, indicating the importance of interview and physical examination. Further investigation is needed to elucidate underlying factors.

Considering the rarity of CS, it is crucial to suspect and diagnose the condition based on clinical symptoms and perform the appropriate screening tests without over- or under-screening [7]. Although CS screening in patients with diabetes mellitus and hypertension has been reported to lead to a diagnosis in only 0–0.7% and 0.1–0.5% of these patients, respectively [38–41], it is ineffective in terms of false positives and cost [9]. Therefore, patients with typical features that are highly specific for CS, such as purple striae, easy bruising, and proximal myopathy [1, 8, 12], as well as those with obesity, diabetes mellitus, or hypertension in combination with these features, should be screened for CS [7, 27]. However, our results suggest that these symptoms are unlikely to be self-recognized. Therefore, the appropriate screening measures must be implemented to establish an early and effective diagnosis of CS.

In these situations, it is crucial for physicians to utilize their knowledge and experience to suspect CS based on symptoms such as typical features [10]. It has been reported that years of clinical experience in endocrine practice can contribute to the estimation of the pre-test probability of CS [16]. In contrast, non-specialists are less likely to encounter patients with CS in their lifetime, which can make it difficult to properly suspect CS [9]. From this perspective, it is of utmost importance that family physicians and general internists are knowledgeable regarding the manifestations that require screening for CS, as early diagnosis of this uncommon and severe condition is crucial [11]. Therefore, it is important for physicians who routinely treat patients presenting with common symptoms such as obesity, diabetes mellitus, and hypertension to meticulously interview and observe for any indicators of CS, even if the patient does not recognize them. Failure to adopt an appropriate tone in these situations may cause the disease to become undetectable.

In rare disorders such as CS, in addition to enhancing public recognition of the disease, the appropriate sharing of information and provision of specialized care in clinical practice remain important issues [42]. Early identification of such rare diseases can be achieved by promoting an understanding of the disease and its symptoms among family, friends, and patients who may be the first to recognize the signs and symptoms in an individual. In fact, in a questionnaire survey of 340 patients with CS across 30 countries, the diagnosis of CS was made in 5.6% of cases by the patients themselves and in 0.9% by their family or friends [43]. In the present study, we found that it took more than 3 years to diagnose CS in 58% of the cases. If CS and its symptoms are popularized among the public, the typical features of CS could be more readily reported to physicians and the time to diagnosis might be shorter. Furthermore, a primary care physician who is well-educated and knowledgeable is crucial in ensuring that the concerns of such individuals are not overlooked.

This study has some limitations. First, this single-center retrospective study included a relatively small sample size with few male patients. Second, CD and ACS have different pathologies; therefore, the frequencies of several CS-related manifestations will differ depending on their subtypes [3, 44]. However, in this study, there was no difference in the discrepancies between patient-reported and physician-assessed manifestations in patients with CD or ACS. Nonetheless, it is crucial that comprehensive research is conducted in larger patient populations with a focus on employing methods that accurately reflect the pathophysiology of CD and ACS. Third, patient reports may be inaccurate in terms of onset and duration because they depend on the patient’s memory. Fourth, the endocrinologists who examined the patients differed, which may have affected the presence or absence of physician-assessed manifestations. Finally, this study investigated the differences between the manifestations reported by patients and those assessed by endocrinologists, although the evaluations conducted by primary care physicians, which are crucial for the early detection of CS, were not available. Future research is needed to investigate the differences in recognizing manifestations between non-specialist physicians and endocrinologists with extensive experience in CS and to examine the changes before and after education for these non-specialists to determine if they can lead to earlier diagnosis of CS.

In conclusion, endocrinologists have been shown to be aware of CS-related symptoms, especially typical features, whereas patients do not recognize these manifestations, even when the disease is severe. Therefore, the key to the early diagnosis and treatment of CS is a more proactive approach of questioning and examining patients suspected of having the disease.
